# The role of dislocation-induced scattering in electronic transport in Ga_x_In_1-x_N alloys

**DOI:** 10.1186/1556-276X-7-490

**Published:** 2012-08-31

**Authors:** Omer Donmez, Mustafa Gunes, Ayse Erol, Cetin M Arikan, Naci Balkan, William J Schaff

**Affiliations:** 1 Science Faculty, Department of Physics, Istanbul University, Vezneciler, Istanbul 34134, Turkey; 2 School of Computer Science and Electronic Engineering, University of Essex, Colchester, Essex CO4 3SQ, United Kingdom; 3 Department of Electrical and Computer Engineering, Cornell University, Ithaca, NY 14853, USA

**Keywords:** Ga_x_In_1-x_N, In-rich Ga_x_In_1-x_N, Mobility, Electronic transport, 72.10.Fk, 72.20.Fr

## Abstract

Electronic transport in unintentionally doped Ga_x_In_1-x_N alloys with various Ga concentrations (*x* = 0.06, 0.32 and 0.52) is studied. Hall effect measurements are performed at temperatures between 77 and 300 K. Temperature dependence of carrier mobility is analysed by an analytical formula based on two-dimensional degenerate statistics by taking into account all major scattering mechanisms for a two-dimensional electron gas confined in a triangular quantum well between Ga_x_In_1-x_N epilayer and GaN buffer. Experimental results show that as the Ga concentration increases, mobility not only decreases drastically but also becomes less temperature dependent. Carrier density is almost temperature independent and tends to increase with increasing Ga concentration. The weak temperature dependence of the mobility may be attributed to screening of polar optical phonon scattering at high temperatures by the high free carrier concentration, which is at the order of 10^14^ cm^−2^. In our analytical model, the dislocation density is used as an adjustable parameter for the best fit to the experimental results. Our results reveal that in the samples with lower Ga compositions and carrier concentrations, alloy and interface roughness scattering are the dominant scattering mechanisms at low temperatures, while at high temperatures, optical phonon scattering is the dominant mechanism. In the samples with higher Ga compositions and carrier concentrations, however, dislocation scattering becomes more significant and suppresses the effect of longitudinal optical phonon scattering at high temperatures, leading to an almost temperature-independent behaviour.

## Background

In the last decade, after the revision of the band gap energy from 1.9 to approximately 0.7 eV [1], intensive research has been carried out on InN and In-rich Ga_x_In_1-x_N alloys in order to re-determine the fundamental properties [2-4]. Despite much interest on the optical properties of InN and Ga_x_In_1-x_N [5,6], there has been a relatively small number of investigations to explain temperature-dependent electronic transport properties in Ga_x_In_1-x_N alloys [7,8].

In this article, we report the electronic transport properties of nominally undoped Ga_x_In_1-x_N alloys with different Ga concentrations (*x* = 0.06, 0.32 and 0.52). Hall effect results show that all the alloys are highly n-type, and the free carrier concentrations are independent of temperature.

## Methods

### Experimental details

The samples with different Ga concentrations (*x* = 0.06, 0.32 and 0.52) were grown by a Varian GEN-II gas source molecular beam epitaxy chamber on (0001) c-sapphire substrates with a 200-nm-thick GaN buffer layer. The growth temperature was varied from low to high with increasing Ga composition [9,10]. The thickness of the Ga_x_In_1-x_N layer was determined from the growth parameters and verified by backscattering spectrometry at nearly 500 nm. The Ga_x_In_1-x_N samples were fabricated in Hall-bar geometry, and ohmic contacts were formed by diffusing Au/Ni alloy. Hall effect measurements were carried out at temperatures between 77 and 300 K.

### Modelling of carrier mobility

The temperature dependence of carrier mobility is analysed using an analytic model where all possible scattering mechanisms are individually calculated using the material parameters given in Table 1. Experimental mobility curves are fitted with the theoretical mobility curves that are obtained using the analytical expressions for the major scattering mechanisms given in Table 2. Although Ga_x_In_1-x_N layer is thick enough (500 nm) not to be two-dimensional (2D), the analytic model considers transport in a 2D electron gas (2DEG). This is because the electronic transport takes place at the interface of Ga_x_In_1-x_N/GaN [11] and on 2D Ga_x_In_1-x_N surface layer [12].

**Table 1:** **The material parameters used in scattering calculations (adopted from [**[10]**,**[13-15]**])**

**Parameter**	**InN**	**GaN**	**Ga**_**x**_**In**_**1-x**_**N**
High-frequency dielectric constant	$\varepsilon_{\infty }=8.4$	$\varepsilon_{\infty }=5.5$	$\varepsilon_{\infty }=8.4-2.9x$
Static dielectric constant	$\varepsilon_{s}=15.3$	$\varepsilon_{s}=8.9$	$\varepsilon_{s}=15.3-6.4x$
Electron effective mass	$m^{*}=0.11m_{0}$	$m^{*}=0.22m_{0}$	$m^{*}=\left(0.1+0.12x\right)m_{0}$
LO-phonon energy	73meV	92meV	$\left(73+11.3x+12x^{2}\right)\text{meV}$
LA-phonon velocity	$v_{s}=5.17\;.10^{3}\;{\text{ms}}^{-1}$	$v_{s}=6.59\;.10^{3}\;{\text{ms}}^{-1}$	$v_{s}=\left(5.17+1.42x\right).10^{3}\;{\text{ms}}^{-1}$
Density of crystal	$\rho =6.81\;.10^{3}\;{\text{kgm}}^{-3}$	$\rho =6.15\;.10^{3}\;{\text{kgm}}^{-3}$	$\rho =\left(6.81-0.7x\right).10^{3}\;{\text{kgm}}^{-3}$
Electron wave vector at Fermi level	$k_{F}=4.61\;.10^{8}\;m^{-1}$	$k_{F}=7.3\;.10^{8}\;m^{-1}$	$k_{F}=\left(7.3+2.69x\right).10^{8}\;m^{-1}$
The electromechanical coupling coefficient	$K^{2}=0.028$	$K^{2}=0.038$	$K^{2}=\left(0.028+0.01x\right)$
Lattice constants	$a=3.53310^{-10}\;m$$c=5.69310^{-10}\;m$	$a=3.189.10^{-10}\;m$$c=5.185.10^{-10}\;m$	$a=\left(3.533-0.344x\right).10^{-10}\;m$$c=\left(5.693-0.508x\right).10^{-10}\;m$
Occupied volume by an atom	Ω0=34a2c	Ω0=34a2c	Ω0=34a2c
Deformation potential	Ξ=7.1eV	Ξ=8.3eV	$\Xi =\left(7.1-1.2x\right)\text{eV}$
Alloy potential	_−_	_−_	$U_{A}=2.72x10^{-19}\;V$

LA-phonon, longitudinal acoustic phonon; LO-phonon, longitudinal optical phonon.

**Table 2:** The formulas of major scattering mechanisms used in 2DEG mobility calculations

**Scattering mechanism**	**Formula**	**Definition of variables**
Acoustic phonon: piezoelectric [15-17]	$\mu_{\text{PE}}=\frac{\pi \varepsilon_{s}\hbar^{3}k}{eK^{2}k_{B}Tm^{*2}}\frac{1}{J_{\text{PE}}\left(k\right)}$	*K*, electromagnetic coupling coefficient; *J*_PE_(*k*), electron wave vector dependent integral.
	$J_{\text{PE}}\left(k\right)=\int_{0}^{2k}\frac{F_{11}\left(q\right)}{4k^{2}{\left(q+q_{s}\right)}^{2}\sqrt{1-{\left(q/2k\right)}^{2}}}q^{3}dq$	
	$K^{2}=\frac{\varepsilon_{\text{LA}}^{2}}{\varepsilon_{s}c_{\text{LA}}}+\frac{\varepsilon_{\text{TA}}^{2}}{\varepsilon_{s}c_{\text{TA}}}$	
Acoustic phonon: deformation [11,18] potential	$\mu_{\text{DP}}=\frac{16\rho ev_{s}^{2}\hbar^{3}}{3\Xi^{2}k_{B}Tm^{*2}b}\frac{1}{J_{\text{DP}}\left(k\right)}$	*ρ*, crystal density; *v*_*s*_, longitudinal acoustic phonon velocity; Ξ_,_ deformation potential constant; m*, electron effective mass; *J*_DP_(*k*), electron wave vector dependent integral. *b*, Fang-Howard expression; *q*_s_, reciprocal screening length; *f*(*0*), occupation probability; *F*_11_(*q*), ground-state Fang-Howard wave function.
	$J_{\text{DP}}\left(k\right)=\int_{0}^{2k}\frac{1}{2k\pi^{3}{\left(q+q_{s}\right)}^{2}\sqrt{1-{\left(q/2k\right)}^{2}}}q^{4}dq$	
	$q_{s}=\frac{e^{2}m^{*}}{2\pi \hbar^{2}\varepsilon_{s}}F_{11}\left(q\right)f\left(0\right)$	
	$b={\left(\frac{33e^{2}m^{*}n_{2D}}{8\varepsilon_{s}\hbar^{2}}\right)}^{1/3}$	
	$F\left(q\right)=b\left(8b^{2}+9qb+3q^{2}\right)/8{\left(b+q\right)}^{3}$	
Polar optical phonon [17-19]	$\mu_{\text{PO}}=\frac{4\pi \varepsilon_{\text{s}}\hbar^{2}}{e\omega m^{*2}Z_{0}}\left[e^{\hbar \omega_{\text{LO}}/k_{B}T}-1\right]$	$\hbar \omega_{\text{LO}}$, polar optical phonon energy; $\varepsilon_{\infty }$ and $\varepsilon_{s}$, high- and low-frequency dielectric constant; *Z*_0_, effective width of triangular well formed at the Ga_x_In_1-x_N/GaN interface and is given in terms of Fermi wave vector.
	$\frac{1}{\varepsilon_{P}}=\frac{1}{\varepsilon_{\infty }}-\frac{1}{\varepsilon_{s}}$	
	$Z_{0}=\frac{2\pi }{k_{F}}=\sqrt{\frac{2\pi }{n_{2D}}}$	
Interface roughness [11,15,20]	$\mu_{\text{IFR}}={\left(\frac{2\varepsilon_{s}}{n_{2D}\Delta \Lambda }\right)}^{2}\frac{\hbar^{3}}{e^{3}m^{*2}}\frac{1}{J_{\text{IFR}}\left(k\right)}$	*Δ*, lateral size of the roughness; _*Λ*,_ correlation length between fluctuations; *J*_IFR_(*k*), correlation length and the lateral size-dependent integral; *n*_2D_, 2D electron density.
	$J_{\text{IFR}}\left(k\right)=\int_{0}^{2k}\frac{\exp\left(-q^{2}\Lambda^{2}/4\right)}{2k^{3}{\left(q+q_{s}\right)}^{2}\sqrt{1-{\left(q/2k\right)}^{2}}}q^{4}dq$	
	$q=2k\sin\left(\theta /2\right)$	
	$q_{s}=\frac{e^{2}m^{*}}{2\pi \varepsilon_{s}\hbar^{2}}F\left(q\right)$	
Alloy disorder [20]	$\mu_{\text{Alloy}}=\frac{16e\hbar^{3}}{3bx\left(1-x\right)m^{*2}\Omega_{0}U_{A}^{2}}$	*x*, Ga fraction; *Ω*_0_, the volume occupied by one atom; *U*_A_, alloy potential.
Dislocation [21-23]	$\mu_{\text{Dis}}=\frac{30\sqrt{2\pi }\varepsilon^{2}c^{2}{\left(k_{B}T\right)}^{3/2}}{e^{3}N_{\text{Dis}}f^{2}\lambda_{D}\sqrt{m^{*}}}$	*N*_Dis_, dislocation density per unit area which is taken as a fitting parameter; *λ*_D_, Debye screening length; *c*, lattice constant of Ga_x_In_1-x_N. *f*, the fraction of filled traps that are assumed fully occupied.
	$\lambda_{D}={\left(\varepsilon_{s}k_{\text{B}}T/e^{2}n_{2D}\right)}^{1/2}$	

## Results and discussions

### Experimental results

**Figure 1:**
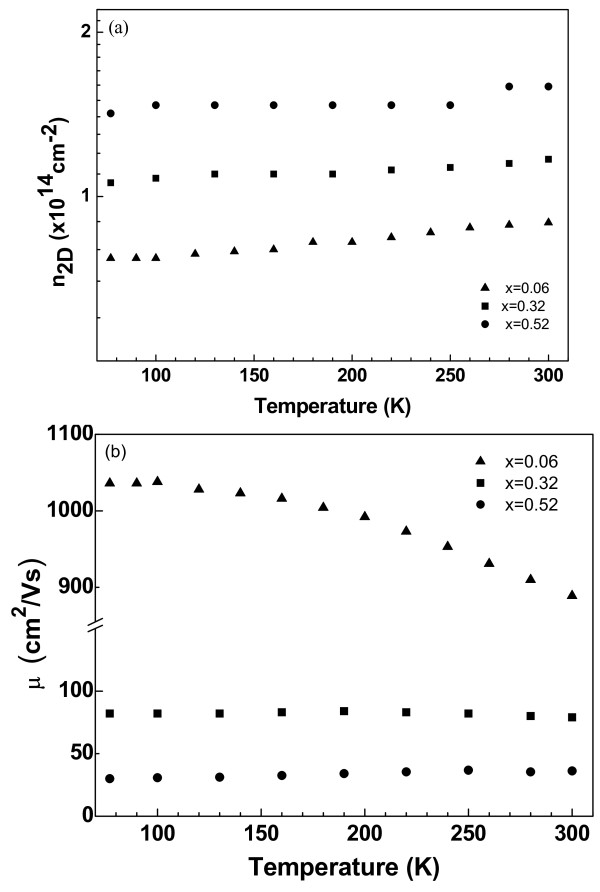
Temperature dependence of (a) carrier density and (b) electron mobility.

Figure 1 shows the temperature dependence of the carrier concentration and the electron mobility between 77 and 300 K for all the samples investigated. Although the samples are not intentionally doped, the Hall effect results show that all the samples have n-type conductivity, and the free carrier densities are independent of the temperature; therefore, samples can be regarded as metallic-like over the whole temperature range as commonly reported by us and by other research groups [7,8,24-28]. It is clear from Figure 1a that the free carrier concentration increases by about a factor of 3 when the Ga composition increases from *x* = 0.06 to 0.52. Also, as seen in Figure 1b, when Ga concentration increases from *x* = 0.06 to 0.52, electron mobility has a sharp decrease from 1,035 cm^2^/Vs for Ga_0.06_In_0.94_ N to 30 cm^2^/Vs for Ga_0.52_In_0.48_ N at 77 K that may be associated with the contribution of both dislocations and point defects in the structure, which are acting as a source of donor-like defects, inducing high electron concentration. In the low-temperature region (≤100 K), the mobility is almost independent of temperature for all the samples. However, for the sample with the lowest Ga concentration, Ga_0.06_In_0.94_ N, it decreases from 1,035 to 890 cm^2^/Vs with increasing temperature from 100 to 300 K but does not show any significant change in the other two samples, which is a characteristic feature of metallic-like semiconductors [7,26,27]. The insensitivity of carrier mobility to temperature is commonly observed in polar materials with elevated carrier densities where the polar interactions are screened [19,25,29-33].

### Modelling of temperature dependence of mobility

In order to understand fully the temperature dependence of electron mobility, we compared the experimental mobility results with analytical theoretical models by taking into account all the possible scattering mechanisms. At low temperatures, the dominant scattering mechanism in bulk semiconductors is ionized impurity scattering that changes with temperature as T^3/2^. However, this kind of temperature dependence has not been observed in our samples. The samples have metallic-like characteristics, confirming the formation of a high-density 2DEG at both the GaN/Ga_x_In_1-x_N interface and on the Ga_x_In_1-x_N surface [26,27]. The dominant momentum relaxation mechanism is the electron-optical phonon scattering in Ga_x_In_1-x_N since it is a highly polar material above T > 150 K [34-36].

In the theoretical calculation, interface roughness, alloy, dislocation, optical and acoustic phonon scattering mechanisms with the appropriate expressions given in Table 2 were considered. The lateral size of the interface roughness *Δ*, correlation length *Λ* between interface fluctuations and the dislocation density are used as adjustable fitting parameters, and the values for the best fit are given in Table 3. The values that we used for the dislocation densities are in good agreement with the transmission electron microscopy (TEM) results taken from Ga_0.34_In_0.66_ N [9,25]. Look et al. [25] determined the dislocation density for both InN and Ga_0.34_In_0.66_ N using TEM and found that dislocation density in Ga_0.34_In_0.66_ N is actually higher than that of InN. It can be seen that the trend of the dislocation density depending on Ga concentration follows the carrier concentration, which means that there is a correlation between dislocation density and the corresponding carrier concentration.

**Table 3:** The values of the parameters used in the calculations

**Sample**	***Δ *(nm)**	***Λ *(nm)**	**Dislocation density (×10**^**10**^ **cm**^**−2**^**)**
Ga_0.06_In_0.94_ N	3.6	1.4 (four monolayer)	0.1
Ga_0.32_In_0.68_ N	6.4	3.4 (ten monolayer)	0.3
Ga_0.52_ In_0.48_ N	6.7	3.4 (ten monolayer)	3.8

**Figure 2:**
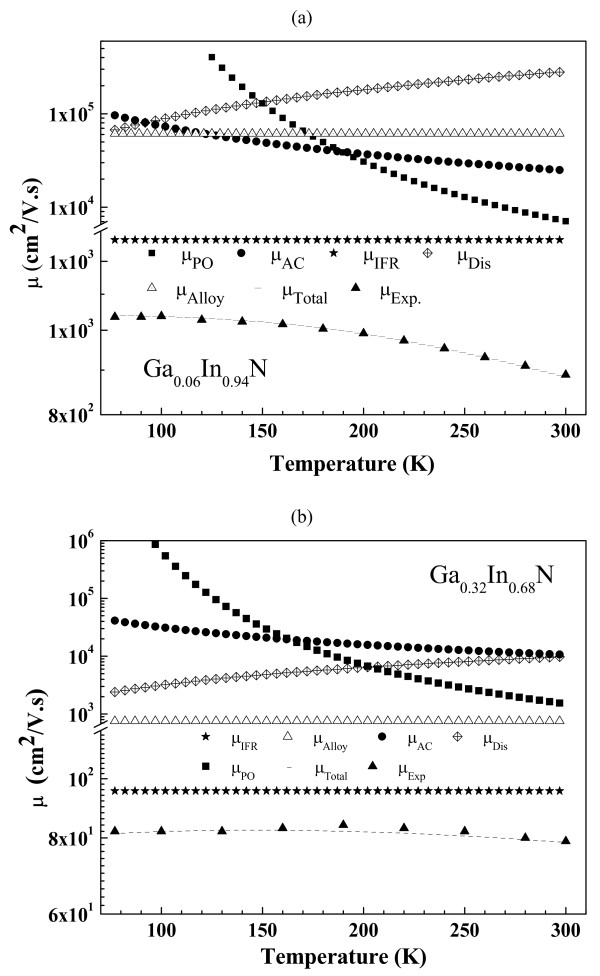
**Experimental and calculated temperature dependence of mobility curves for (a) Ga**_**0.06**_**In**_**0.94**_ **N and (b) Ga**_**0.32**_**In**_**0.68**_ **N.**

**Figure 3:**
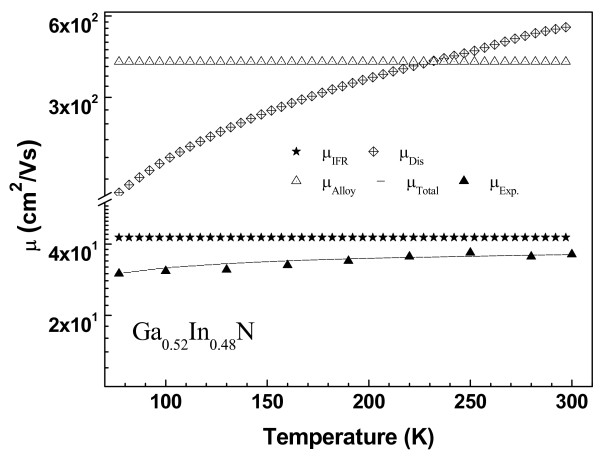
**Measured and calculated mobility versus temperature Ga**_**0.52**_**In**_**0.48**_ **N.**

It is clear from Figure 2 that at low temperatures, electron mobilities in Ga_0.06_In_0.94_ N and Ga_0.32_In_0.68_ N are determined by alloy potential-induced scattering, interface roughness scattering and dislocation scattering mechanisms. Optical phonon scatterings become significant at high temperatures, as described above. Figure 3 shows experimental and calculated temperature-dependent mobility of the Ga_0.52_In_0.48_ N. The dislocation density increases with Ga concentration; therefore, its effect on the mobility becomes more pronounced in this sample. At low temperatures, mobility is limited by the same scattering mechanisms as in the other samples. At high temperatures, however, interface roughness and alloy potential restrict the mobility, but effect of the dislocation scattering becomes less dominant as a result of shortening Debye screening length due to higher carrier density. Furthermore, in the high-carrier-concentration regime, electron–phonon scattering is heavily screened, as described above and in references [19,25,29-33].

## Conclusions

In this paper, we have investigated electronic transport properties of nominally undoped In-rich Ga_x_In_1-x_N structures with different Ga concentrations. Hall effect results show that 2DEG mobility in Ga_x_In_1-x_N decreases and becomes temperature insensitive with increasing Ga concentrations. The samples are not intentionally doped, but they all have n-type conductivity. Electron density increases with increasing Ga composition. The temperature dependence of electron mobility is determined by taking into account all the major scattering mechanisms. The decrease of the electron mobility with Ga concentration is explained in terms of increased dislocation scattering. The weak temperature dependence of the mobility at high temperatures might be associated with reduced electron-optical phonon scatterings. Alloy and interface roughness scattering mechanisms are dominant at low temperatures. In samples with higher Ga fractions, dislocation scattering becomes more significant, and at high temperatures, phonon scattering is restricted due to increase of dislocation density. At high temperatures, phonon scattering is only pronounced in the samples with low electron densities.

## Abbreviations

LO-phonon: longitudinal optical phonon; LA-phonon: longitudinal acoustic phonon; 2DEG: two-dimensional electron gas; TEM: transmission electron microscopy; IFR: interface roughness.

## Competing interests

The authors declare that they have no competing interest.

## Authors' contributions

OD and MG carried out the experiments and fitted the Hall mobility data with AE and MCA. OD, MG, AE and MCA wrote the manuscript in conjunction with NB. WJS grew the investigated samples. All authors read and approved the final manuscript.
